# A signal processing based analysis and prediction of seizure onset in patients with epilepsy

**DOI:** 10.18632/oncotarget.6341

**Published:** 2015-11-17

**Authors:** Hamidreza Namazi, Vladimir V. Kulish, Jamal Hussaini, Jalal Hussaini, Ali Delaviz, Fatemeh Delaviz, Shaghayegh Habibi, Sara Ramezanpoor

**Affiliations:** ^1^ School of Mechanical and Aerospace Engineering, Nanyang Technological University, Singapore; ^2^ Faculty of Medicine, Universiti Teknologi MARA (UiTM), Selangor, Malaysia; ^3^ Department of Otolaryngology, Faculty of Medicine, Shiraz University, Shiraz, Iran; ^4^ Faculty of Mechanical Engineering, Iran University of Science and Technology, Tehran, Iran; ^5^ Faculty of Chemical Engineering, Islamic Azad University (Fars Science and Research Branch), Shiraz, Iran; ^6^ School of Medicine, Fasa University of Medical Sciences, Fasa, Iran

**Keywords:** epileptic seizure, prediction, EEG signals, the Hurst exponent, fractal dimension

## Abstract

One of the main areas of behavioural neuroscience is forecasting the human behaviour. Epilepsy is a central nervous system disorder in which nerve cell activity in the brain becomes disrupted, causing seizures or periods of unusual behaviour, sensations and sometimes loss of consciousness. An estimated 5% of the world population has epileptic seizure but there is not any method to cure it. More than 30% of people with epilepsy cannot control seizure. Epileptic seizure prediction, refers to forecasting the occurrence of epileptic seizures, is one of the most important but challenging problems in biomedical sciences, across the world. In this research we propose a new methodology which is based on studying the EEG signals using two measures, the Hurst exponent and fractal dimension. In order to validate the proposed method, it is applied to epileptic EEG signals of patients by computing the Hurst exponent and fractal dimension, and then the results are validated versus the reference data. The results of these analyses show that we are able to forecast the onset of a seizure on average of 25.76 seconds before the time of occurrence.

## INTRODUCTION

An epileptic seizure is a brief episode of signs and/or symptoms due to abnormal excessive or synchronous neuronal activity in the brain. More than 50 million people worldwide suffer from epilepsy [1]. Seizure symptoms can vary widely. Some people with epilepsy simply stare blankly for a few seconds during a seizure, while others repeatedly twitch their arms or legs. Seizures have a beginning, middle, and end. Not all parts of a seizure may be visible or easy to separate from each other. Every person with seizures will not have every stage. The epileptic seizure can have external or internal reasons. Sometimes an external stimulus can cause the onset of a seizure. On the other hand, an internal stimulus can cause the degrees of freedom of the inherent brain dynamical system to get reduced, which takes the brain to an abnormal status. The recurrent and sudden incidence of seizures can cause dangerous and possibly life-threatening situations [2].

Since disturbance of consciousness and sudden loss of motor control often occur without any warning, the ability to predict epileptic seizures would reduce patients' anxiety, thus improving quality of life and safety considerably. In this light, in the absence of completely controlling a patient's epilepsy, seizure prediction is an important aim of clinical management and treatment.

During years different methods have been developed for prediction of epileptic seizure. Electroencephalogram is one of the important tools for diagnosis and analysis of epilepsy. In fact, Electroencephalography changes preceding seizures can theoretically be detected to permit anticipation of oncoming seizures.

In order for EEG-based seizure forecasting systems to work effectively, computational algorithms must reliably identify periods of increased probability of seizure occurrence.

The first EEG-based attempts at identifying preictal patterns relied primarily on linear approaches for computing features of the EEG on a sliding window [3–4]. These models gave way to nonlinear signal processing methodologies, which analyzed the spontaneous formation of spatial, temporal, and spatiotemporal patterns [5].

Various features have been computed from EEG time series in order to detect changes immediately prior to the onset of seizures. These include some of the more traditional frequency-based methods discussed below, as well as more recent measures derived from complex system theory.

In case of linear feature extraction, employing some methods such as Principal Components Analysis (PCA) [6–8], auto regressive spectral analysis [9–10], Support Vector Machine [11–13], are noteworthy to mention.

On the other hand some researchers have focused on using nonlinear methods/measures such as phase synchronization analysis [14–16], Kolmogorov entropy [17], Lyapunov exponents [18–20], correlation dimension [21–22], approximate entropy [23–24], Dynamical Similarity Index [25–26], and permutation entropy [27–28].

Having some advantages and disadvantages common between these methods, in many of these methods EEG analysis is complicated by the fact that EEG manifestations of seizures differ widely between patients and even within the same patient. Also, methods employed in seizure prediction are mathematically complex and not easily accessible to those outside of the world of physicists, mathematicians, and engineers. So, these phenomena strongly suggest that the continued research is needed in this area.

In this research we consider the reduction in the degrees of freedom of the inherent brain dynamical system due to the internal stimuli which causes the epileptic seizure. In fact, for a patient with epilepsy who is seated without receiving any external stimulus, the presence of a strong internal stimulus is the main reason of seizure onset. So, by studying the EEG signal prior to onset and finding the dominant sign of stimulus we can predict the coming seizure.

In the following, first we focus on introducing two measures, the Hurst exponent and fractal dimension. Then by applying these measures to EEG signals of patients with epilepsy we try to find the sign of a coming seizure by studying the variations of these measures.

## EEG TIME SERIES

**Figure 1:**
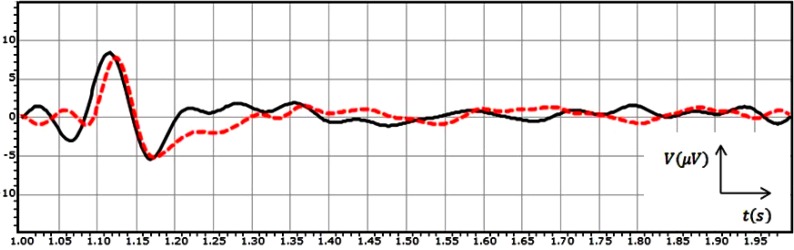
The grand average of the recorded EEG signals from two subjects for 1 second post-stimulation in the case of the visual stimulus

During many years scientists have studied the human behaviour by recording and analysis of EEG signals from different areas of the brain. The EEG signal is the composition of different frequency bands (oscillatory activities-Alpha, Beta, …) which are structured coordinately (spatially-temporally). In fact, this signal has different characteristics that can be used in order to study the human brain response to external or internal stimuli. For instance Figure 1 shows the grand average of the recorded EEG signals from two subjects for 1 second post-stimulation in case of the visual stimulus.

As it can be seen in Figure 1, the EEG signal for subject 1 (black solid line) and the EEG signal for subject 2 (red dashed line) shows the similar behaviours. For subject 1 and subject 2 the response to the stimulus starts with a positive peak (P) at 118 ms and 127 ms respectively after the application of the stimulus to the subjects. This response causes the signal's voltage fluctuates in a bigger span. Following the positive peak a negative rebound (N) at *t* = 1.170 s and *t* = 1.174 s can be seen in the plot in cases of two signals respectively. In fact, the response to the stimulus damped at this point, after which the brain goes back to its normal status during rest, without any big deflection in the signal.

## HURST EXPONENT AND PREDICTABILITY OF SIGNAL

The Hurst exponent is a measure of the predictability of signal. It is an indicator of the long term memory of the process generating the signal. The Hurst exponent can have any value between 0 and 1, where the value that it gains in each moment determines the behaviour of the next deflection in the signal.

**Figure 2:**
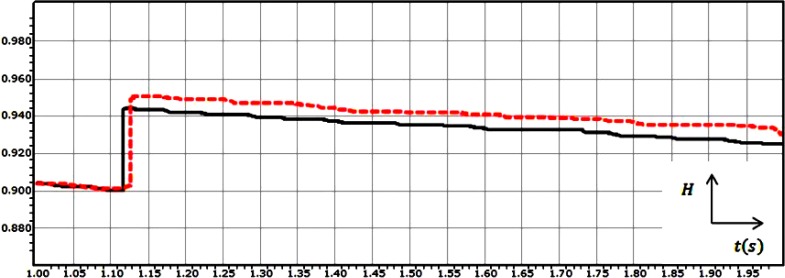
The grand average of the Hurst exponent variations for the recorded EEG signals from two subjects for 1 second post-stimulation in the case of the visual stimulus

Firstly, if the Hurst exponent has a value between 0 and 0.5, it means that the process is anti-persistent i.e. the trend of the process at the next instant will be opposite to the trend in the previous instant. Secondly, a value of H between 0.5 and 1 means that the process is persistent i.e., the trend of the process at the next instant will be the same as the trend in the previous instant. Finally, If H = 0.5, the process is considered to be truly random (e.g., Brownian motion). It means that there is absolutely no correlation between values of the process. Figure 2 shows the grand average of the Hurst exponent variations for the recorded EEG signals which was shown in Figure 1.

The high correlation between the values of the EEG signal for subject 1 and also EEG signal for subject 2 can be realized by looking at the values of the Hurst exponents. The value of the Hurst exponent is distributed between 0.900 and 0.943 for subject 1 and between 0.900 and 0.950 for subject 2.

Also, as it can be seen in the Hurst exponent plot, the value of the Hurst exponent in case of subject 1 and subject 2 experiences a sudden upward deflection. In case of subject 1 and subject 2, the value of the Hurst exponent is decreasing in the time span of *t* = 1 s to *t* = 1.118 s and *t* = 1 s to *t* = 1.127 s respectively; After that, a sudden upward deflection can be seen, which stands for experiencing the visual stimulus and increasing the memory, and again the trend shows the same behaviour. The overall decreasing behaviour stands for the phenomenon that when a longer time span is considered, the less the human brain “remembers” its initial state [29].

## SPECTRA OF FRACTAL DIMENSION

In this section we explain the fractal dimension as the measure of complexity of the fractal time series, and in our case here the EEG signals [30].

The concept of fractal dimension is based on the concept of generalized entropy of a probability distribution, introduced by Alfred Renyi [31]. In case of a time series with V_max_ and V_min_, where the total range of the value is divided into N bin:

$$N=\frac{V_{\text{max}}-V_{\text{min}}}{\delta V}$$

The probability that the value falls into the *i*-th bin of size δV is computed as:

$$w_{i}=\lim_{N\to \infty }\frac{N_{i}}{N}$$

where N_*i*_ equals the number of items the value falls into the *i*-th bin. On the other hand, in case of a time series:

$$w_{i}=\lim_{T\to \infty }\frac{t_{i}}{T}$$

Where *t_i_* is the time spent by the value in the *i*-th bin during the total time span of recording, T.

Starting with the moment of order *q* (not necessarily an integer) of the probability *w_i_*, the Renyi entropy is:

$$E_{q}=\frac{1}{1-q}log_{2}\sum_{i=1}^{N}w_{i}^{q}$$

Note that for *q* → 1, [32]:

$$E_{1}=-\sum_{i=1}^{N}w_{i}\;log_{2}w_{i}$$

The generalized fractal dimensions of a given time series with the known probability distribution are defined as:

$${ℵ}_{q}=\lim_{\delta \;V\to 0}\frac{1}{q-1}\frac{{\text{log}}_{2}\sum_{i=1}^{N}w_{i}^{q}}{{\text{log}}_{2}\delta V}$$

Where the parameter *q* ranges from − ∞ to + ∞. Note that for a self-similar (simple) fractal time series with equal probabilities *w_i_* = 1/N, equation (6) yields ℵ_*q*_ = ℵ_0_ for all values of *q* [33]. Also, note that for a constant value, all probabilities except one become equal to zero, whereas the remaining probability value equals unity.

For a given time series (‘signal’), the function ℵ_*q*_, corresponding to the probability distribution of the series, is called the fractal spectrum. Such a name is well-justified, because the fractal spectrum provides information about both frequencies and amplitudes of the series. Indeed, for two probability distributions, a larger value of fractal dimension of a given order corresponds to the presence of more pronounced spikes (sharper spikes, less expected values of the signal) than in the series for which the value of the fractal dimension of the same order is less. Furthermore, series with a wider range of fractal dimensions, ℵ_−∞_ −ℵ_∞_, can be termed more fractal than series whose range of fractal dimensions is narrower, so that series with the zero range are self-similar (simple) fractals. In other words, the range of a fractal spectrum is a value associated with the range of frequencies in the series.

Now, if the unexpectedness of an event is defined as the inverse of the probability of this event, then steeper spectra correspond to the series in which unexpected values are more dominant, whereas flatter spectra represent those series in which less unexpectedness occurs [30].

## RESULTS AND DISCUSSION

Here, in order to investigate about the onset of seizures, the EEG records and their Hurst exponent and fractal dimension plots are studied for subjects with epilepsy. A MATLAB based program was written in order to compute these parameters. The outcomes are discussed in details.

### Data collection

In this research the EEG data were collected from 120 patients with epilepsy, 60 male patients and 60 female patients with the age of 25 ± 5 years old. None of the patients had received medication before their recruitment. In the first week 5 trials were collected from each subject in one day. The data collections were repeated after a week for each subject in order to examine the reproducibility of the results from experiments. By repeating the experiments in the second week totally 10 trials were collected. After visual inspection of data collected from each subject and rejection of trials with artifacts, 8 trials free of artifacts were selected for future analysis. It is noteworthy to mention that physician monitored the subjects during all experiments.

Informed consent was obtained from each subject after the nature of the study was fully explained.

All procedures were approved by the Internal Review Board at Nanyang Technological University and the approval for the experimentation on human subjects with epilepsy was issued by this university and the hospital. It is noteworthy to mention that the identity of all subjects remains confidential.

The subjects were asked to sit in an electrically shielded, acoustically isolated, and dimly illuminated room. This ensures that the response measured in the EEG signals doesn't have any external stimulus source. It should be mentioned that it is endeavoured to insulate the subjects from all other external stimuli.

The EEG data used in this research were collected using Mindset 24 device, a 24-channel topographic neuro mapping instrument, which can measure 24 channels of data with the sampling frequency of 256 Hz. Mindmeld 24 software was used for the collection of data using Mindset 24 machine. The software gives data in the form of .bin files which can be processed to give text files (.txt) that are required for further processing.

Although the EEG data are recorded from 24 electrodes, in this research the analysis is done on the data governed from the electrode with biggest fluctuations to have a clear view of the seizure. In fact this electrode shows the strongest response of brain.

In order to filter the artifacts, we did the band pass filtering of the EEG data at the frequency of 35 HZ, and 3 minutes of data (6300 data) was saved. It means that there are 35 values of voltage every second.

### Data analysis

As it was mentioned, in order to compute the Hurst exponent and fractal dimension, a MATLAB based program was written. Computation of fractal dimension is based on the equations discussed before. In case of the Hurst exponent computation, there are different methods which have been developed to estimate the value of H. Rescaled Range Analysis (R/S) and DFA are two mostly used methods of the Hurst exponent estimation. By the initial analysis of the computed the Hurst exponent of EEG time series we found out that even if R/S method shows higher values of the Hurst exponent than DFA, the standard deviations are lower for R/S so that the confidence intervals are narrower and thus in our case R/S method is more precise. Nevertheless, we found out that both methods show similar results which become closer as the EEG time series becomes longer. So in this research we employ R/S analysis method for computing the Hurst exponent. It is noteworthy that the initial value of H is computed for 5 minutes of the recorded data. As it was mentioned, in this research we analyzed the EEG data for 120 subjects, but here we only discuss the result of analysis of two subjects in details. Other results are provided in Table 1.

**Table 1:** The difference time between the sign of seizure in the Hurst exponent and fractal dimension plots, and its onset for 120 subjects

No	Value	No	Value	No	Value	No	Value	No	Value	No	Value
1	30	21	29	41	27	61	20	81	24	101	20
2	20	22	29	42	29	62	28	82	27	102	19
3	26	23	32	43	24	63	22	83	20	103	27
4	25	24	22	44	30	64	26	84	32	104	26
5	19	25	28	45	31	65	29	85	21	105	20
6	22	26	27	46	26	66	20	86	22	106	31
7	32	27	32	47	25	67	30	87	29	107	32
8	27	28	19	48	20	68	19	88	27	108	30
9	23	29	22	49	23	69	23	89	27	109	27
10	29	30	27	50	27	70	27	90	26	110	24
11	19	31	29	51	20	71	34	91	34	111	30
12	26	32	21	52	31	72	24	92	19	112	33
13	28	33	35	53	32	73	20	93	24	113	19
14	20	34	32	54	25	74	22	94	29	114	19
15	21	35	29	55	25	75	26	95	25	115	25
16	23	36	23	56	29	76	30	96	24	116	29
17	28	37	26	57	19	77	31	97	22	117	20
18	34	38	30	58	20	78	26	98	20	118	31
19	27	39	27	59	32	79	24	99	19	119	25
20	28	40	22	60	27	80	27	100	29	120	27

**Figure 3:**
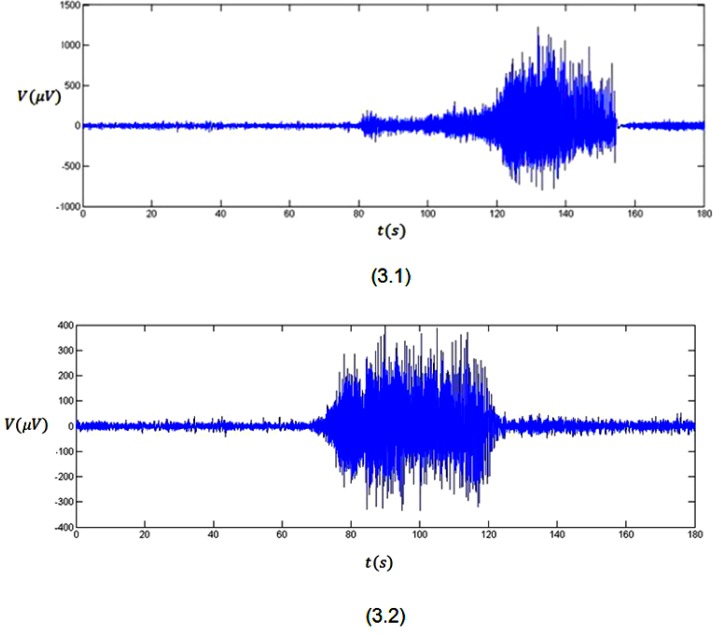
Three minutes recorded EEG signals from two subjects

Plots 3.1 and 3.2 in Figure 3 show the average of three minutes recorded EEG signals with pre-seizure, seizure and some post-seizure activity for two subjects with epilepsy, who is resting without receiving any external stimulus. By looking at plot 3.1, it is clear that the brain has its normal activity from *t* = 0 s to about *t* = 80 s (pre-seizure) when the EEG voltage falls in the normal range of variation. The seizure is clear in the plot from about *t* = 80 s to about *t* = 160 s (seizure). In this time span, the EEG voltage fluctuates between −769 μV to 1230 μV. Also, this plot shows some post-seizure activity after *t* = 160 s till *t* = 180 s when the brain goes back to its normal status. The mentioned behaviour also can be seen in plot 3.2. In this plot the pre-seizure is from *t* = 0 s to about *t* = 70 s. The seizure is clear in the plot from about *t* = 70 s to about *t* = 120 s. Also, this plot shows some post-seizure activity after *t* = 120 s till *t* = 180 s when the brain goes back to its normal status.

**Figure 4:**
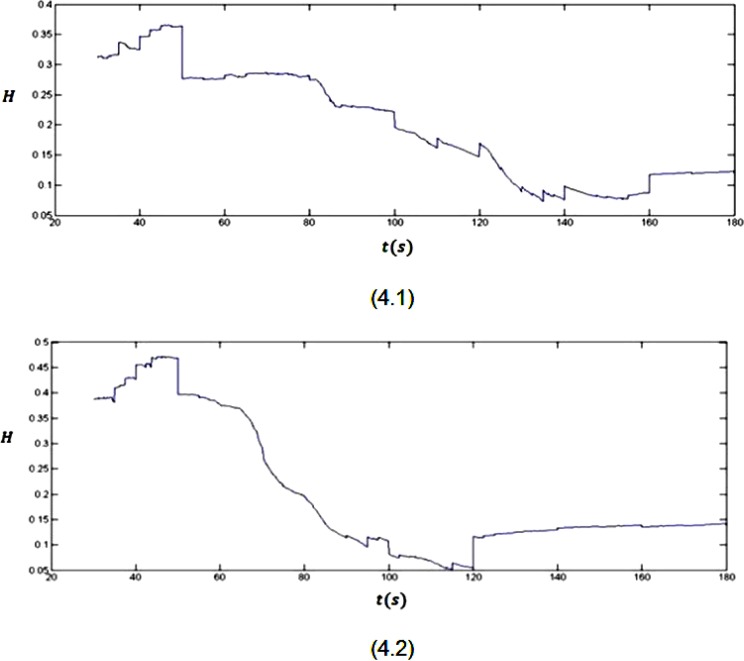
The Hurst exponent variations for three minutes recorded EEG signals from two subjects

As it was mentioned before, the effect of a stimulus (bigger than threshold value) on the brain is mapped as a sudden deflection in the Hurst exponent plot. This sudden deflection temporarily takes the values of the Hurst exponent far from 0.5 (H = 0.5 stands for a truly random process). Using this phenomenon and looking at the Hurst exponent plots (Figure 4) for the EEG signals (Figure 3), the onset of seizure can be discussed.

As it is clear in plot 4.1, a sudden downward deflection at about *t* = 50 s can be seen. Considering the subject is resting without receiving any external stimulus, the mentioned sudden deflection can only be related to an internal stimulus. In fact, this stimulus later, at about *t* = 80 s, causes the brain to have an abnormal activity (epileptic seizure). After this stimulus till the end of seizure (*t* = 50 s to *t* = 160 s), the value of the Hurst exponent decreases from 0.5. But, because these values are between 0 and 0.5, a good correlation between the values of the signal cannot be seen. When the brain goes back to its normal status (after *t* = 160 s), a sudden upward deflection can be seen in the plot, which increases the values of the Hurst exponent. Although this upward deflection sometimes is small, but it is the beginning of the process which increases the values of the Hurst exponent. As it was mentioned previously, this behaviour stands for the phenomenon that when a longer time span is considered, the less the human brain “remembers” its initial state. The mentioned behaviour also can be seen in plot 4.2. In this plot the seizure onset is clear by the downward deflection at *t* = 50 s. The seizure ends by the upward deflection at *t* = 120 s in this plot.

**Figure 5:**
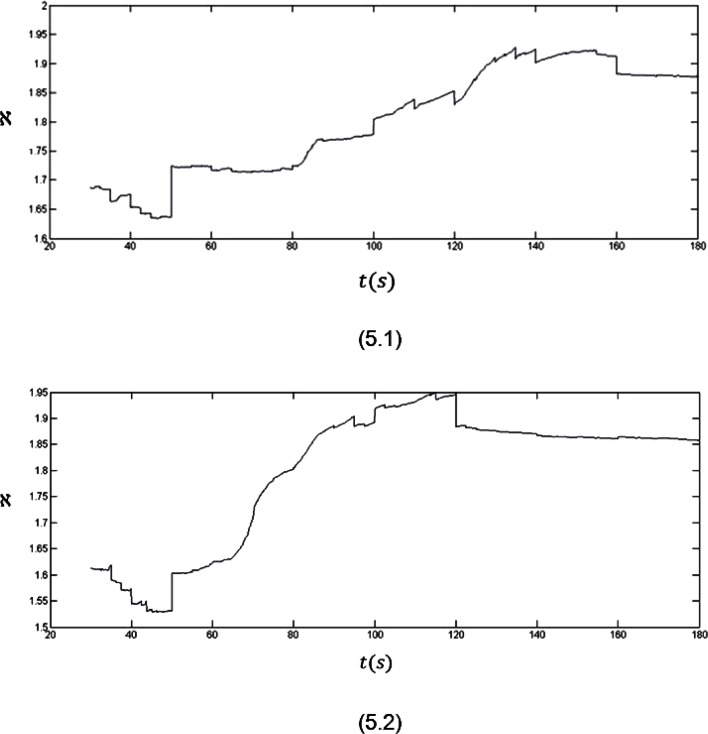
The fractal dimension variations for three minutes recorded EEG signals from two subjects

In order to show the strength of our analysis the fractal dimension variations for the EEG records are shown in Figure 5.

The analyses of the fractal dimension plots for the record of EEG signals give results which do not deviate from what has been observed in the Hurst exponent plots.

As it is clear in plot 5.1, a sudden upward deflection at about *t* = 50 s can be seen in the fractal dimension plot which is related to an internal stimulus. In fact, the internal stimulus makes the EEG signal more complex as the values of the fractal dimension start to increase. After this stimulus till the end of the seizure (*t* = 50 s to *t* = 160 s), the values of the fractal dimension increase. As it can be seen in the plot, when the brain goes back to its normal status (after *t* = 160 s), a sudden downward deflection can be seen in the plot, where the values of the fractal dimension start to decrease and so less complexity will be seen in the signal.

The mentioned behaviour also can be seen in plot 5.2. In this plot the seizure onset is clear by the upward deflection at *t* = 50 s. The seizure ends by the downward deflection at *t* = 120 s in this plot.

So, it can be said that analysing the fractal dimension plots can certify the strength of our claim discussed above.

The same analyses have been done on other subjects. The Hurst exponent and fractal dimension plots in all cases show the similar behaviour and could predict the seizure onset. Table 1 list the time difference between the sign of seizure in the Hurst exponent and fractal dimension plots, and its onset for all patients. As the difference between the sign of seizure and its onset is same in case of the Hurst exponent and Fractal dimension plots, here we only report one value for it. The value in each case is the average of values related to eight measurements for each subject. The results indicate that the sign of seizure was predicted at least 19 seconds before the onset (in case of subjects 5, 11, 28, 57, 68, 92, 99, 102, 113 and 114). The average of all cases is 25.76 second.

**Figure 6:**
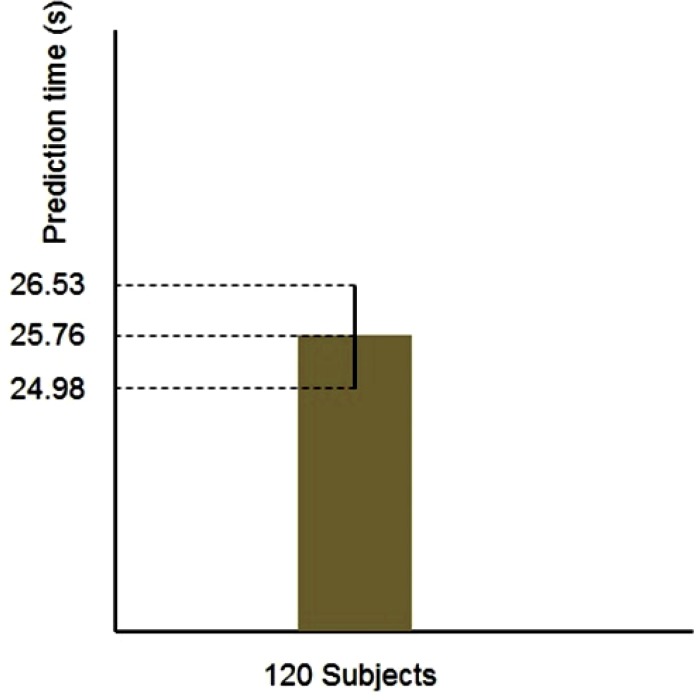
Confidence interval for the time difference between the sign of seizure and its onset (standard deviation = 4.30)

Also, in order to compare the time difference between the sign of seizure in the Hurst exponent and fractal dimension plots and its onset in case of different subjects, we compute 95% confidence interval for samples population. Figure 6 shows the computed confidence interval.

As it is clear in this figure, confidence interval has the variation range of 24.98 ≤ *X* ≤ 26.53. This narrow range indicates that the governed value of 25.76 s stands for the average of all samples with high confidence.

So, by analysing the Hurst exponent and fractal dimension plots the sign of seizure onset was seen for all patients. Thus, our analysis showed that we can predict the seizure onset before the time of occurrence.

## CONCLUDING REMARKS

In this research, we proposed a new methodology which predicts the onset of epileptic seizure by analysing the Hurst exponent and fractal dimension plots of the EEG records. The results of analyses for 120 patients showed that forecasting of the seizure is possible an overage 25.76 seconds before its onset. This unique methodology can be helpful in analysis and prediction of other abnormal activities of the brain. Also this methodology can be used in developing a portable hand-held device which records the EEG signals from the patient's brain using a scalp and further analyses the signal by the described methodology in this research. So, the device can give the alert to patients before the seizure onset, thus patient can take the required medication in order to prevent the seizure.
